# Metric Goodness and Measurement Invariance of the Italian Brief Version of Interpersonal Reactivity Index: A Study With Young Adults

**DOI:** 10.3389/fpsyg.2021.773363

**Published:** 2021-12-20

**Authors:** Pierluigi Diotaiuti, Giuseppe Valente, Stefania Mancone, Angela Grambone, Andrea Chirico

**Affiliations:** ^1^Department of Human Sciences, Society and Health, University of Cassino, Cassino, Italy; ^2^Department of Psychology of Development and Socialization Processes, Sapienza University of Rome, Rome, Italy

**Keywords:** IRI, empathy, prosocial behavior, confirmatory analysis, convergent validity, gender invariance, alexithymia

## Abstract

The Interpersonal Reactivity Index (IRI) is a widely used multidimensional measure to assess empathy across four main dimensions: perspective taking (PT) empathic concern (EC) personal distress (PD) fantasy (F). This study aimed to replicate the Italian validation process of the shortened IRI (Interpersonal Reactivity Index) scale in order to confirm its psychometric properties with a sample of young adults. The Gender Measurement Invariance of empathy in this age group was also an objective of the work in order to increase the data on this aspect. A total of 683 Italian university students participated in a non-probabilistic sampling. The 16-item version was confirmed in its four-factor structure but with changes to some items. The model showed good fits with both the CFA and the gender Measurement Invariance. The internal consistency measures were found to be fully satisfactory. Convergent validity was tested by the correlations with the *Prosocialness Scale for Adults* and *The Toronto Alexithymia Scale-20*. As hypothesized the measure proved good convergent validity with Prosocialness, i.e., the willingness to assist, help, share, care and empathy with others, and a relevant inverse association with the External Oriented Thinking, characterizing individuals with emotionally poor thinking. This research provided additional evidence for a link between alexithymia and poor empathic abilities in young adults.

## Introduction

Empathy refers to the ability to share and understand another person's thoughts and moods (Decety and Moriguchi, 2007). Feeling the emotion of others, understanding it fully is also essential to guide one's actions in a pro-social sense and especially to avoid implementing those behaviors that can cause damage and suffering in the other. Empathy is first of all an individual characteristic and belongs to the genetic background of the individual, but it also reflects the thought system of the person that includes their values, beliefs and motivations that are learned from education and experience of their lives.

Empathy cannot exist unless there is emotional involvement, but it can only be considered a fundamental element of pro-social behavior when cognitive processes are put in place. The feeling of empathy can in fact vary from a less evolved stage, called “contagion” to increasingly refined forms of awareness and differentiation (Caprara, 2006). The awareness of emotions would develop through different evolutionary stages, from a purely sensorimotor level, to a formal operational level, characterized by a full awareness of emotions and the ability to perform operations of differentiation of the different emotional nuances of one's own and others' emotions.

The first authors to present an integrated model of empathy were Feshbach and Feshbach (1987), who referred to empathy as a process that integrates the cognitive component, as a prerequisite, and the affective component, as a determinant of empathy. Their research showed that the two empathic components should be regarded as complementary rather than opposing elements. The action of all the components together would make it possible to perform an integrated action capable of generating empathy. This theory proposes that empathy is composed of three essential competences or skills, of which the first two are cognitive skills and only the last reflects the affective dimension.

More specifically, these abilities are: decoding the emotional states experienced by other people (knowing how to recognize and classify the emotions of others); assuming the role and perspective of another (knowing how to put oneself in the place of the other, taking on the other person's point of view, even if it is different from one's own); responding affectively to the emotions felt by another person (being able to share in a vicarious manner the emotions of others). According to Feshbach's model, empathy does not appear until the overcoming of egocentrism around the age of 6.

Hoffman's model (Hoffman, 1987), unlike the one just presented, proposes that empathy arises from the first days of life, when the affective component is accentuated compared to the cognitive abilities that instead will acquire a greater and progressive importance over time. According to this theory, cognitive abilities intertwine with affective components over time and this leads to increasingly evolved forms of empathy. In addition to the two components mentioned above, Hoffman's model adds a further element, namely, the motivational component. The author proposes that the act of empathy toward a person who is suffering is a motivation to engage in pro-social behavior. This motivation would derive from the fact that empathizing with the other person leads us to lend him or her support and this, in turn, leads to a feeling of well-being for those who act in this way; conversely, not helping the other person would lead to a sense of guilt. Thus, empathy is not a static skill, but rather a skill that changes and evolves over time and goes along with cognitive skills.

Hoffman identifies five ways in which empathy manifests itself: (1) *Global empathic distress*, present in the first months of life, when one is unable to distinguish between oneself and the other. Thus, an emotion of another becomes one's own, but one is not able to understand that the cause comes from the emotional state of the other. Empathy in this early stage of life takes the form of a totally affective, involuntary and automatic response. (2) *Egocentric empathic distress*, developed during the first year of life, the child begins to distinguish the other from himself but is not yet able to distinguish internal states from those of others. The child acts by imitating the emotions of others and may make attempts to support the other, which should not be understood as altruistic as they are aimed at attenuating his emotional state. (3) *Quasi-egocentric empathic distress*, takes shape during the first and second year of life when the distinction from the other is clearer, behaviors are set in motion to compare the other, such as the act of embracing him, but the egocentric component remains. In fact, when one acts to compare the other one chooses to do actions that would be meaningful for oneself. (4) *True empathy for another person's state of mind* begins around the age of two when one begins to be aware of the distinction between the internal states and the emotional states of others. As a result, the child no longer acts according to how he or she feels but according to the wishes and feelings of the other person. (5) *Empathic distress beyond the situation*, takes place from the age of nine, when the child realizes his or her own identity and its influence in different life situations. Full or more mature empathy is reached around the age of thirteen when the complexity of cognitive processes is high enough.

Eisenberg and Strayer (1987) considers empathy, like Hoffman, from an evolutionary and multidimensional perspective. Similarly, she considers that the development of cognitive components is progressively integrated with affective components and this leads to the establishment of more evolved forms of empathy. On the contrary, according to the author, emotional contagion, which Hoffman calls global empathic distress, is an automatic and involuntary affective response. In order to speak of empathy, cognitive mediation processes must be involved. This is why the model does not take into account the vicarious affective sharing typical of the first years of life. Only two forms of empathy are considered: (1) *Empathy by parallel sharing*, in which rudimentary cognitive processes are involved and which takes place in pre-school age. It describes a process whereby when one observes an event that affects another, one tends to bring to mind one's own experience that is similar to that event, and to bring into being the emotion one experienced at that moment. (2) *Transient sharing empathy*, which is the most mature form of empathy and is mediated by more evolved and complex cognitive mechanisms, develops when the child is able to understand that the other, having his or her own identity, might experience different emotions from those he or she would experience in the same circumstances.

Davis (1980) with his theoretical construct highlights an aspect of empathy that had not been taken into account by other models. According to him, empathy can sometimes lead to acts of altruism, but it also has a dark side, so that very often people act only in order to protect themselves from the discomfort caused by the emotional state of others. Specifically, his approach identifies four components of the empathic response: the ability to adopt the other's point of view (perspective taking), the ability to imagine fictional situations (fantasy), the tendency to experience feelings of compassion and concern toward the other (empathic concern) and finally, the awareness of one's own states of anxiety experienced in relational situations (personal distress). The latter two are the emotional components of empathy and represent two different ways of approaching the other's situation. In particular, personal distress is characterized by a selfish motivation because observing the suffering of others creates in the observer a state of anxiety and consequently he acts in favor of the other but with the aim of freeing himself from the state of distress he experiences in first person. On the contrary, empathic concern is characterized by an altruistic motivation, the observer shares the emotional states of others and sets in motion pro-social behaviors aimed essentially at improving the conditions of the other.

Comparing the different models with a muldimensional approach, the original characterization of the measure of Davis' Personal Distress made us consider the use of this model preferable in the assessment of adult empathy. Davis (1980) has indeed developed a specific tool for its measurement, the Interpersonal Reactivity Index (IRI), divided into 28 items, in turn grouped into four subscales (Perspective Taking, Fantasy, Empathic Concern, Personal Distress). The characteristics of the multidimensional approach and its psychometric properties have made the IRI the most commonly used instrument to detect empathic responsiveness among adults.

The IRI was initially administered to a large number of college students (1,161 subjects), demonstrating good internal consistency (Cronbach's alpha ranges from 0.70 to 0.78). Davis (1983) demonstrated the validity of the instrument by comparing the relationship between the four subscales and some potentially related constructs: interpersonal functioning/social competence, self-esteem, emotionality, and sensitivity to others. The intelligence measure was also included for informational purposes. Next, he assessed relationships between the IRI and an affective measure (Mehrabian and Epstein, 1972) and a cognitive measure of empathy (Hogan, 1969). The PT subscale was associated with good interpersonal functioning and high self-esteem, whereas there were no significant relationships with the dimensions of emotionality and sensitivity to others, which were associated with the EC subscale.

The PT subscale had a strong correlation with the cognitive measure of empathy and the EC subscale with the affective measure. High scores in the PD subscale were associated with reduced self-esteem and low interpersonal functioning, as well as an emotional picture characterized by vulnerability and uncertainty. The F subscale showed a similar pattern to the EC subscale, its relationship with measures of emotional reactivity being stronger. By comparing males and females, Davis showed higher scores for the latter in all four subscales, especially in the Fantasy subscale. The cognitive subscale Perspective Taking presents the least differences between the sexes. The author then analyzed the correlations between the subscales, which were significant, although modest. The most important relationship concerns the positive link between PT, EC and F. The PT subscale shows an inverse relationship with the PD subscale. The associative pattern between the two subscales with emotional content, EC and PD, was not significant.

Numerous studies, conducted subsequently, have reaffirmed what was already illustrated by Davis: four-factor structure; satisfactory relationships among subscales, particularly between PT and EC; good relationships between empathy and pro-social behavior and negative relationships between empathy and indices of violent and antisocial behavior (Franzoi et al., 1985; Carey et al., 1988; Riggio et al., 1989; Burke, 2001; Lindsey et al., 2001; Pérez-Albéniz et al., 2003).

The IRI has been used as a tool to explore the personality correlates of caregivers who serve in helping settings (physicians, nurses, and volunteers) showing that high scores on the Perspective Taking and Empathic Concern subscales are predictive of greater emotional availability to the suffering of others, while high scores on the Personal Distress subscale are indicators of a strong level of burn-out (Day and Chambers, 1991; Yarnold et al., 1996; Unger and Thumuluri, 1997).

The scale has been used to investigate traumatic brain injury patients' abilities to assess or detect emotional stimuli (McLellan and McKinlay, 2013; Saxton et al., 2013). The IRI has also been widely used in psychiatry and neuroscience, particularly in the study of personality disorders, as well as schizophrenia (Chiang et al., 2014; Fujino et al., 2014; Lehmann et al., 2014; Michaels et al., 2014).

The IRI questionnaire has been adapted into other languages, including Spanish (Pérez-Albéniz et al., 2003), German (Enzmann, 1996; Lauterbach and Hosser, 2007), Dutch (De Corte et al., 2007), Chinese (Siu and Shek, 2005), Swedish (Kulich and Bengtesson, 2002), Korean (Kang et al., 2009), and French (Gilet et al., 2013).

Alterman et al. (2003) suggested a three-factor model that resulted from a confirmatory factor analysis conducted on the IRI administered to a population of adult drug-addicted patients on methadone treatment. On the first factor - called general empathy - it saturated a combination of items originally placed in the Empathic Concern and Perspective Taking subscales, while on the second and third factors it saturated some items from the Fantasy subscale and others from the Personal Distress subscale, respectively.

Poulos et al. (2004) identified a second-order two-factor structure: the first corresponded to the concept of empathy and contained items from the Empathic Concern and Perspective Taking subscales; the second, called “emotional control,” was positively associated with the Perspective Taking subscale and negatively with the Personal Distress subscale.

In Italy, the study conducted by Albiero et al. (2006) involved administering the IRI to a group of 828 adolescents (47% male), aged between 10 and 20 years (mean age = 14.75 years, ds = 2.34). The analyses conducted confirmed the four-factor organization hypothesized by Davis, the patterns of correlation between the various subscales, and the psychometric characteristics of the instrument (1980, 1983, 1994). Scale reliability was satisfactory with good internal consistency. Finally, gender and age were relevant factors in explaining individual differences in empathic ability. The administration of the IRI to physicians, nurses, and volunteers also provided additional support for the above theses: high scores on the PT and EC subscales are indicative of a greater emotional predisposition to the suffering of others, whereas high scores on the PD subscale point toward a strong level of detachment (Yarnold et al., 1996; Unger and Thumuluri, 1997).

To address some problems, including some uncertainties about its factorial structure, low reliability, and poor readability of some items for people with limited reading skills, Ingoglia et al. (2016) proposed an initial shortened form of the index, the Brief IRI (B-IRI). Three studies here demonstrated that the 16-item B-IRI had consistent factorial structure, adequate internal consistency, measurement invariance across gender and age, and theoretically meaningful associations with a number of external criteria supporting its construct validity.

The present study aims to provide additional evidence on the metric goodness of the Italian Brief version of the IRI, reproducing the validation process starting from Davis' original version with 28 items, and also evaluating the convergence in association with different measures than those employed by Ingoglia et al. (2016). We believe that the use of an exclusively adult sample and not of adolescents may require further adaptation of the brief instrument to make it more reliable in measuring the four dimensions of the model in this age group. There are currently no further studies in the Italian psychometric literature that have used the brief version of the IRI with young adults.

Convergent validity was assessed through a direct association with prosocial behaviors and feeling empathic toward others, and an expected inverse association with alexithymia as construct indicating deficiency in the interpretive and evaluative component of affect. In connection with this it was therefore hypothesized that the higher the *IRI* scores, the higher the prosocial behavious and the lower the alexithymia would have been. Since in Davis' model the person's prosocial response is modulated by the active empathic component (Empathic Concern connoted by an altruistic motivation vs. Personal Distress connoted by a selfish motivation), in the study the convergence pattern is assessed with a short unidimensional Italian instrument that specifically measures the prosocial disposition in adults (Prosocialness Scale for Adults, Caprara et al., 2005). Caprara himself emphasizes that in adulthood a person's empathic motivations or predispositions are not simply a correlate of their tendency to act in a prosocial manner but, rather, an integral part of this tendency. For the analysis of convergence with alexithymia the TAS-20 instrument was chosen, which in the Italian validation (Bressi et al., 1996) was administered to both clinical and non-clinical samples of adult subjects. This scale is suggested for both clinical and research use. Ultimately, the study also proposes testing for gender invariance of the instrument. Measurement invariance is fundamental in psychological research because it is a prerequisite for comparing group averages (Putnick and Bornstein, 2016). The topic of gender differences in people's empathic capacity has been the subject of numerous studies showing greater empathy in females than males (Carlo et al., 1999; Baron-Cohen and Wheelwright, 2004; Mestre et al., 2009; Carrasco et al., 2011). Some have reported that gender differences may have a biological basis, as evidenced by endocrine and neural correlates associated with gender differences observed on self-report empathy measures (Fukushima and Hiraki, 2006; Singer et al., 2006; Rueckert and Naybar, 2008), others also emphasized the value of role expectations (Löffler and Greitemeyer, 2021). The current study also aimed to investigate whether the measure of the brief IRI would be gender invariant with a sample of young adults, and what differences can be found when considering the four dimensions of the measurement model, thus increasing the observational data on this aspect.

## Method and Materials

### Participants and Procedure

The overall sample used for the study consisted of 300 university students, coming from the center regions of Italy, of whom 142 were male (47.3%) and 158 female (52.7%), aged between 18 and 31, with an average age of 22 years (SD = 2.63). They freely took part in the study after completing and signing the form for informed consent to participate. Participants covered a substantially equal number of students attending science (47%) and humanities courses (53%). They were administered the Italian version of *Interpersonal Reactivity Index* (Albiero et al., 2006), composed of 28 items with Likert scale 1 (never true)-5 (always true). Davis reported a four-factors structure: (1) Perspective taking (PT), assesses attempts to adopt the perspectives of other people; (2) Fantasy (F), assesses the tendency to identify with fictional situations; (3) Empathic Concern (EC), assesses whether the individual has the tendency to experience compassionate feelings toward people in distress; and (4) Personal Distress (PD), assesses the personal feelings of discomfort when the individual is observing another's negative experiences or is facing distressing situations. The administration of the instruments was done through an on-site group procedure. The average compilation time was about 8 min.

The short version of the scale obtained through the CFA on data from the previous administration was then included in a new protocol to test the convergent validity of this tool. For this purpose was used an additional convenient sample of participants (*N* = 383), of whom 163 were male (42.6%) and 220 female (57.4%), with had an average age of 23 years (SD = 5.9). Also in this case participants covered a substantially equal number of students attending science (44%) and humanities courses (56%). The inclusion criterion in this case was non-participation in the previous administration. They were therefore administered in succession: (1) the Brief version of *Interpersonal Reactivity Index*, 16 items distributed over four factors: the first contains five items, the second contains four, and the fourth and fifth contain three each. Every item has a scoring range from 0 (does not describe me at all) to 5 (it describes me completely). The person is asked to assess what he/she thinks and feels in different situations and to indicate the answer that best describes him/her. The scoring calculation will produce separate measurements for each factor, through a summation of the scores of the component items, and a total Index score corresponding to the sum of the subscale scores. Therefore, *Perspective Taking*: 1 + 5 + 9 + 13 + 14; *Personal Distress*: 2 + 6 + 10 + 15; *Fantasy*: 3 + 7 + 11; *Empathic Concern*: 4 + 8 + 12+ 16. The score for the items 4,8 and 12 should be reversed. (2) the *Prosocialness Scale for Adults* (PSA, Caprara et al., 2005) composed of 17 items and classifies behaviors and feelings into four types: the action of assisting, helping, sharing of caring and empathy with others. The tool assesses adults rating of their own sharing, helping, taking care of, and feeling empathic toward others (e.g., “I try to help others”) on a 5-point Likert scale ranging from 1 (never or almost never true) to 5 (almost always or always true). (3) *The Toronto Alexithymia Scale-20* (TAS-20, Bagby et al., 1994; Bressi et al., 1996), a 20-item self-report instrument with each item rated on a 5-point Likert scale ranging from 1 (strongly disagree) to 5 (strongly agree); five items are inversely rated. Total scores range between 20 and 100, and higher scores mean a higher tendency toward alexithymia. The tool consists of three factors: (1) seven items for difficulty in identifying feelings and distinguishing them from the bodily sensations of emotions (DIF); five items for difficulty in describing feelings (DDF); eight items to measure the tendency of individuals to focus their attention externally (EOT). In this case too the administration of the instruments was done through an on-site group procedure, while the total compilation time was about 18 min.

### Statistical Analysis

The sample size was determined by the ability to confirm a satisfactory fit of IRI starting with a four-factor model with 28 manifest variables. When utilizing the root-mean-square error of approximation (RMSEA) as a measure of model fit, a minimum of 280 participants provides a 90% power level to test RMSEA 0.05 when RMSEA = 0.08 (MacCallum et al., 1996).

The following were the main statistical analyses performed: verification of univariate and multivariate normality assumptions; Confirmatory Factorial Analysis (CFA); assessment of internal consistency using Cronbach's alpha coefficient and McDonald's omega (Cronbach, 1951, 2004; McDonald, 1999). The main advantage of using McDonald's omega rather than Cronbach's a when evaluating the quality of short-scale measures is that reliability estimated using coefficient omega does not increase or decrease with the number of items in the scale. The evaluation of significance of correlation coefficients was implemented to test concurrent validity of the tool. The Composite Reliability Index (CRI) was used to investigate reliability: it's regarded good if the value is more than 0.70 (Raykov, 1997).

The following 10 metrics were used to assess the model's adequacy: (1) chi square; (2) the relationship between the chi-square value and the degrees of freedom (χ^2^/d.f., values between 1 and 3 are considered acceptable); (3) GFI (*Goodness of Fit Index*), with values higher than 0.90 indicating an acceptable fit of the model, while a good fit with values higher than 0.95; (4) RMSEA (*Root-Mean-Square Error of Approximation*), with values between 0.05 and 0.8 indicating an acceptable fit of the model, while a good fit with values lower than 0.05; (5) *p-value for the test of close fit*, with values between 0.50 and 1 indicating an acceptable fit of the model, while a good fit with values between 0.05 and 0.50; (6) CFI (*Comparative Fit Index*) and TLI (*Tucker-Lewis Index*), with values between 0.95 and 0.97 indicating an acceptable fit of the model, while a good fit with values between 0.97 and 1; (7) NFI (*Normed Fit Index*), with values between 0.90 and 0.95 indicating an acceptable fit of the model, while a good fit with values between 0.95 and 1 (Hu and Bentler, 1999; Byrne, 2001; Schermelleh-Engel et al., 2003; Barbaranelli and Ingoglia, 2013); (8) PNFI (*Parsimony Normed Fit Index*), with values between 0.50 and 0.60 indicating an acceptable fit of the model, while a good fit with values between 0.60 and 1 (Mulaik et al., 1989).

The gender measurement invariance of the *IRI* factorial structure was investigated. Therefore, four nested models with increasing degrees of restriction were tested: the base model assessed configural invariance and allowed free estimation of all the parameters for each group. The metric (weak) invariance model, nested in the configural model, added the restriction of invariant factor loadings among groups. The scalar (strong) invariance model, nested in the second model, added the intercept constraint of the invariant items among the comparison groups. Finally, we tested strict invariance by comparing the scalar model to a model that also constrains residuals to be equal across tested groups. We concentrated on comparing the CFI, TLI, and RMSEA indices because the Chi-square indices are sensitive to sample size. To rule out invariance, we used a variation of these indices >0.01 as a criteria of the more restrictive model and accept the more parsimonious model (Cheung and Rensvold, 2002).

When the strict invariance was verified, the group mean differences in latent variables were tested.

The correlations between the *IRI* variables and the factors that make up PSA and TAS-20 were compared to determine concurrent validity. Pearson coefficients were used to determine concurrent validity. SPSS version 22, JASP 0.12.2, and IBM Amos Graphics 18 were used to conduct statistical analysis.

## Results

In order to obtain a brief version of the scale, factor loadings from *IRI* (28 items) were considered, selecting among them the items with loadings > 0.50. Sixteen items were extracted, whose descriptive statistics are presented in Table 1.

**Table 1:** Descriptive statistics of the Interpersonal Reactivity Index (IRI) (*N* = 300).

**Item**	**M**	**SD**	**Bootstrap CI 95%**	**SK**	**KU**
Item 4	3.79	1.10	(3.66–3.92)	−0.668	−0.430
Item 6	3.11	1.05	(2.99–3.23)	−0.291	−0.577
Item 8	3.56	1.01	(3.44–3.68)	−0.497	−0.120
Item 9	3.93	0.86	(3.83–4.03)	−0.751	0.586
Item 10	2.86	1.03	(2.74–2.97)	0.044	−0.587
Item 11	3.76	0.82	(3.66–3.85)	−0.639	0.817
Item 14	3.60	1.08	(3.46–3.73)	−0.432	−0.577
Item 16	3.13	1.01	(3.02–3.26)	−0.054	−0.551
Item 17	3.05	1.05	(2.93–3.17)	0.158	−0.662
Item 18	4.05	1.04	(3.93–4.16)	−01.08	0.582
Item 21	3.71	0.88	(3.62–3.82)	−0.434	0.103
Item 23	3.33	0.93	(3.22–3.44)	−0.250	−0.264
Item 24	2.59	1.01	(2.48–2.70)	0.263	−0.495
Item 25	3.36	0.97	(3.25–3.47)	−0.304	−0.175
Item 26	3.36	0.98	(3.26–3.46)	−0.254	−0.266
Item 27	3.64	0.94	(3.52–3.75)	−0.536	0.215

*M, Mean; SD, Standard Deviation; SK, Skewenes; KU, Kurtosis; CI, Confidence Interval*.

The model with four linked components and 16 items produced overall good indices of data adaption, according to the confirmatory factorial analysis: Chi-square = 117.996; *p* = 0.030; χ^2^/df = 1.296; CFI = 0.975; TLI = 0.968; GFI = 0.953; RMSEA = 0.031 and RMSEA 90% CI (0.010–0.047), PCLOSE = 0.980; NFI = 0.903; PNFI = 0.685.

**Figure 1:**
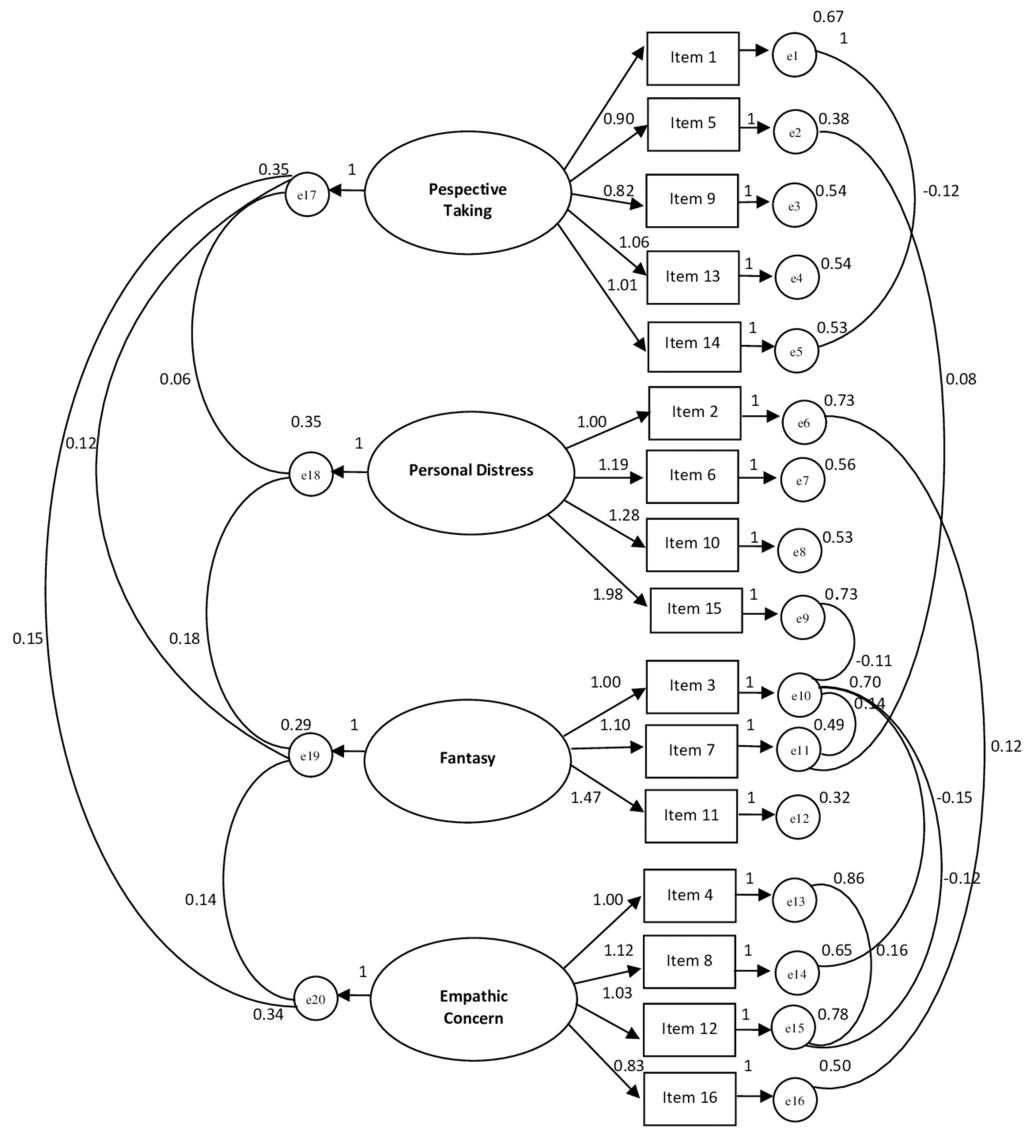
Path diagram of the confirmatory analysis concerning IRI (16 items).χ^2^/df = 1.296; RMSEA = 0.031; RMSEA 90% CI = 0.010–0.047; GFI = 0.953; TLI = 0.968; CFI = 0.975; NFI = 0.903.

As can be seen in Figure 1, the first factor measures *Perspective Taking* (five items); the second factor measures the *Personal Distress* (four items); the third factor measures the *Fantasy* (three items); the fourth factor measures the *Empathic Concern* (four items).

Table 2 shows the model matrix with saturations on the four selected factors, McDonald's ω and Crombach's Alpha values, Guttman Split-Half Coefficients, Corrected item/total correlations, Uniqueness. All factorial loadings were statistically significant (*p* < 0.001) and ranged between 0.549 and 0.765. The CRI (0.727) was good. In Table 3 the factorial interrelatioships are reported.

**Table 2:** Pattern Matrix EFA (16 items).

	**PT**	**PD**	**EC**	**F**	**Uniqueness**
Item 25	0.747	0.036	−0.042	−0.129	0.518
Item 8	0.595	0.016	−0.058	−0.032	0.680
Item 11	0.587	0.053	0.062	0.066	0.565
Item 21	0.580	−0.070	−0.113	0.089	0.674
Item 27	0.548	−0.069	0.107	0.032	0.639
Item 17	0.055	0.735	−0.039	−0.035	0.473
Item 10	−0.069	0.627	−0.009	0.107	0.471
Item 24	−0.081	0.608	−0.095	−0.061	0.543
Item 06	0.078	0.572	0.070	−0.005	0.635
Item 14	−0.050	0.043	0.721	−0.098	0.535
Item 18	−0.067	−0.111	0.662	0.038	0.579
Item 04	0.002	−0.014	0.645	−0.071	0.608
Item 09	0.250	0.018	0.569	0.104	0.667
Item 16	0.025	−0.077	−0.185	0.782	0.488
Item 23	−0.013	0.014	0.016	0.756	0.417
Item 26	−0.032	0.148	0.185	0.569	0.481
***α***	0.68	0.72	0.70	0.75	
95% IC	(0.62, 0.74)	(0.67, 0.77)	(0.64, 0.75)	(0.70, 0.79)	
**Ω**	0.69	0.73	0.71	0.75	
95% IC	(0.64, 0.75)	(0.68, 0.78)	(0.65, 0.76)	(0.70, 0.80)	
***λ6***	0.66	0.67	0.63	0.67	
r*	0.31	0.39	0.36	0.50	

Extraction Method: Maximum Likelihood. Rotation Method: Promax with Kaiser Normalization. Rotation converged in five iterations. Cumulative variance: 56.63%. α, Cronbach's alpha; ω, McDonald's omega; λ6, Gutmann's lamda; r^*^, average inter-item correlation.

**Table 3:** Factor inter-correlations.

	**Prospective taking**	**Personal distress**	**Empathic concern**
Prospective taking	1		
Personal distress	0.197	1	
Empathic concern	0.393	0.096	1
Fantasy	0.414	0.521	0.319

In addition, the measurement invariance of the brief IRI's factorial structure by gender was evaluated. Four nested models with increasing degrees of restriction were tested. Table 4 shows the goodness-of-fit indices of the multidimensional model by gender and nested models of invariance in ascending order of restriction level. The *IRI* brief demonstrated significant gender invariance, and the fit of the four-dimensional model for male and female was outstanding, according to the findings.

**Table 4:** Tested models and goodness-of-fit indices.

	**χ^**2**^**	**df**	**Δχ^**2**^**	**Δdf**	**CFI**	**TLI**	**RMSEA**	**ΔCFI**	**ΔTLI**	**ΔRMSEA**
Models in each group										
Gender										
Male	85.943	76			0.979	0.970	0.030			
Female	84.360	76			0.988	0.984	0.023			
Gender										
Configural	204.078^*^	158	–	–	0.953	0.938	0.044	–	–	–
Metric	222.945^*^	169	18.67	11	0.946	0.932	0.046	−0.007	−0.006	0.002
Scalar	232.325^*^	180	9.380	11	0.947	0.938	0.044	0.001	0.006	−0.002
Strict	253.716^*^	200	21.391	20	0.946	0.943	0.042	−0.001	0.005	−0.002

*df, degrees of freedom; χ^2^, Chi square; Δχ^2^, difference in Chi square; Δdf, difference in degrees of freedom; CFI, comparative fit index; TLI, Tucker-Lewis index; RMSEA, root mean square error of approximation; ΔCFI, difference in comparative fit index; ΔTLI, difference in Tucker-Lewis index; ΔRMSEA, difference in root mean square error of approximation*.

* *p < 0.001*.

These results mean that the latent means can be compared by gender. The latent mean values were fixed to zero for females and, as could be seen in the following Table 5, females showed in this study higher latent mean values of Perspective Taking, Personal Distress, Fantasy, Empathic Concern than men. In particular, significantly higher values of personal distress can be noted in women.

**Table 5:** Group mean differences in latent variables.

**Variables**	**Factors**	**Mean**	**SE**	**CR**	***p***
Gender (male)^*^	F_1_	−0.98	0.21	−4.71	<0.001
	F_2_	−1.15	0.26	−4.46	<0.001
	F_3_	−0.81	0.18	−4.54	<0.001
	F_4_	−0.95	0.25	−3.77	<0.001

* *Reference variable is female; SE, Standard error; CR, Critical ratio; F_1_, Perspective Taking; F_2_, Personal Distress; F_3_, Fantasy; F_4_, Empathic Concern*.

The significance of correlation coefficients with the *Prosocialness Scale for Adults* (PSA, Caprara et al., 2005) and *The Toronto Alexithymia Scale-20* (TAS-20, Bagby et al., 1994; Bressi et al., 1996) was used to examine convergent validity. Convergent validity testing was performed on a new sample: 383 individuals (163 males 42.6% and 220 females 57.4%) with an average age of 23.30 and SD = 5.90. Two hypotheses have been proposed in light of the findings of these associations: (1) the higher the *IRI total scores and its components*, the higher the total *PSA* would have been; (2) the higher the *IRI total scores and its components*, the lower the *total TAS* and its components would have been. The results, as shown in Table 6, corroborated the first hypothesis's correlation directions; while for the second hypothesis, the expected inverse association was found only with the External Oriented Thinking subscale. Therefore, the measure proved good convergent validity with prosocialness, i.e., the willingness to assist, help, share, care and empathy with others.

**Table 6:** Correlations of the interpersonal reactivity index (IRI) with the prosocialness scale for adults [PSA and the toronto alexithymia scale-20 (TAS-20)].

**Variables**	**IRI**	**PSA**	**TAS-20**	**PT**	**PD**	**EC**	**F**	**DIF**	**DDF**	**EOT**
IRI	1									
PSA	0.597^**^	1								
TAS-20	−0.070	−0.213^**^	1							
PT	0.666^**^	0.572^**^	−0.217^**^	1						
PD	0.567^**^	0.150^**^	0.349^**^	0.117^*^	1					
EC	0.590^**^	0.398^**^	−0.277^**^	0.275^**^	0.002	1				
F	0.749^**^	0.433^**^	−0.025	0.423^**^	0.357^**^	0.145^**^	1			
DIF	−0.186^**^	0.024	0.754^**^	−0.067	.−0.428^**^	−0.109^*^	−0.225^**^	1		
DDF	−0.111^*^	0.033	0.764^**^	0.040	−0.283^**^	−0.041	0.021	0.449^**^	1	
EOT	−0.477^**^	−0.554^**^	0.396^**^	−0.461^**^	−0.093	−0.369^**^	−0.315^**^	−0.002	0.076	1

*IRI, Interpersonal Reactivity Index; PSA, Prosocialness Scale for Adults; TAS-20, Toronto Alexithymia Scale-20; PT, Prospective Taking; PD, Personal Distress; EC, Empathic Concern; F, Fantasy; DIF, Difficulty in Identifying Feelings; DDF, Difficulty in Describing Feelings of Others; EOT, Externally Oriented Thinking*.

** *Correlation is significant at the 0.01 level (2-tailed)*.

* *Correlation is significant at the 0.05 level (2-tailed)*.

In Table 7 below, the study measures are shown comparatively by gender, F and η^2^ values. It can be seen that in the females of the sample the values of Empathic Concern and Prosociality are significantly higher, whereas in the males higher values of EOT (Externally Oriented Thinking) emerged.

**Table 7:** Means and standard deviations of the Brief *IRI, PSA, TAS-20* by Gender, F and η^2^ values.

	**Males** **(*****N*** **=** **163)**		**Females** **(*****N*** **=** **220)**			
	**M**	**SD**	**M**	**SD**	**F**_**(1, 382)**_	**η^2^**
Perspective Taking (B-IRI)	3.45	0.64	3.63	0.65	7.51^**^	0.02
Personal Distress (B-IRI)	2.82	0.74	3.08	0.75	11.92^***^	0.03
Empathic Concern (B-IRI)	3.44	0.81	4.03	0.84	47.99^***^	0.11
Fantasy (B-IRI)	3.14	0.75	3.37	0.86	7.42^**^	0.02
Prosocialness Scale for Adults (PSA)	3.41	0.59	3.86	0.56	58.21^***^	0.13
Difficulty Describing Feelings (DIF, TAS-20)	2.63	0.77	2.76	0.86	2.18	0.01
Difficulty Identifying Feeling (DDF, TAS-20)	2.90	0.71	3.02	0.98	1.76	0.005
External Oriented Thinking (EOT, TAS-20)	2.39	0.77	1.99	0.63	31.09^***^	0.07

*** *p < 0.001;*

** *p < 0.01*.

The internal reliability of the two samples used in this study, as well as their confidence intervals, are provided in Table 8. McDonald's ω and Alpha coefficients for these convergent administrations ranged from 0.74 to 0.75 (*Perspective Taking*), from 0.70 to 0.72 (*Personal Distress*), from 0.76 to 0.77 (*Empathic Concern*), from 0.70 to 0.71 (*Fantasy*), respectively.

**Table 8:** Internal reliabilities of the two samples.

	**Sample 1** **(*****N*** **=** **300)**					**Sample 2** **(*****N*** **=** **383)**		
**Variables**	***α***	**C.I**.	**ω**	**C.I**.	***α***	**C.I**.	**ω**	**C.I**.
Perspective taking	0.68	(0.62, 0.74)	0.69	(0.64, 0.75)	0.73	(0.70, 0.76)	0.74	(0.70, 0.77)
Personal distress	0.72	(0.67, 0.77)	0.73	(0.68, 0.78)	0.70	(0.64, 0.76)	0.71	(0.66, 0.78)
Fantasy	0.75	(0.70, 0.79)	0.75	(0.70, 0.80)	0.76	(0.71, 0.79)	0.77	(0.72, 0.80)
Empathic concern	0.70	(0.64, 0.75)	0.71	(0.65, 0.76)	0.70	(0.64, 0.75)	0.71	(0.65, 0.76)
IRI total	0.77	(0.73, 0.81)	0.77	(0.74, 0.81)	0.80	(0.77, 0.83)	0.81	(0.78, 0.84)

*α, Cronbach's alpha; ω, McDonald's omega; C.I., 95 % Confidence Interval*.

Further CFA with the second sample confirmed again the goodness of fit values for this four-factor, sixteen-item model: χ^2^ = 128.852; df = 90; CFI = 0.974; TLI = 0.965; RMSEA = 0.034; RMSEA 90% CI = 0.019–0.046. The English and Italian versions of the IRI-B, as well as the grouping of the items on corresponding factors, are presented in Table 9.

**Table 9:** Interpersonal reactivity index - brief (IRI-B).

**English version**	**Italian version**
1. I try to look at everybody's side of a disagreement before I make a decision (PT).	1. In caso di disaccordo cerco di tener conto del punto di vista di ognuno prima di prendere una decisione.
2. In emergency situations, I feel apprehensive and ill-at-ease (PD).	2. In situazioni di emergenza, mi sento apprensivo e a disagio.
3. After seeing a play or movie, I have felt as though I were one of the characters (F).	3. Dopo avere visto una rappresentazione teatrale o un film, mi sono sentito come se io stesso fossi uno dei protagonisti.
4. Sometimes I don't feel very sorry for other people when they are having problems (EC) (R).	4. A volte non mi sento molto dispiaciuto per altre persone che hanno problemi.
5. I sometimes try to understand my friends better by imagining how things look from their perspective (PT).	5. A volte cerco di comprendere meglio i miei amici immaginando come le cose appaiono dalla loro prospettiva.
6. I sometimes feel helpless when I am in the middle of a very emotional situation (PD).	6. A volte mi sento indifeso quando mi trovo in situazioni emotivamente molto coinvolgenti.
7. When I watch a good movie, I can very easily put myself in the place of a leading character (F).	7. Quando guardo un buon film, riesco molto facilmente a mettermi nei panni di un personaggio principale.
8. When I see someone being treated unfairly, I sometimes don't feel very much pity for them (EC) (R).	8. Quando vedo qualcuno che viene trattato ingiustamente, talvolta mi capita di non provare molta pietà per lui.
9. I believe that there are two sides to every question and try to look at them both (PT)	9. Credo che esistano due opposti aspetti in ogni vicenda e cerco di prenderli in considerazione entrambi.
10. Being in a tense emotional situation scares me (PD).	10. Trovarmi in situazioni che provocano tensione emotiva mi spaventa.
11. When I am reading an interesting story or novel, I imagine how I would feel if the events in the story were happening to me (F).	11. Quando leggo una storia o un racconto interessante, immagino come mi sentirei se gli avvenimenti nella storia stessero accadendo a me.
12. Other people's misfortunes do not usually disturb me a great deal (EC) (R).	12. Le sventure delle altre persone a volte non mi turbano molto.
13. When I'm upset at someone, I usually try to “put myself in his shoes” for a while (PT).	13. Quando sono in contrasto con qualcuno, di solito cerco di “mettermi nei suoi panni” per un attimo.
14. Before criticizing somebody, I try to imagine how I would feel if I were in their place (PT).	14. Prima di criticare qualcuno, cerco di immaginare cosa proverei se fossi al suo posto.
15. I tend to lose control during emergencies (PD).	15. Tendo a perdere il controllo in caso di emergenza.
16. When I see someone being taken advantage of, I feel kind of protective toward them (EC).	16. Quando vedo qualcuno che viene sfruttato, provo sentimenti di protezione nei suoi confronti.

*PT, Perspective Taking; PD, Personal Distress; EC, Empathic Concern; F, Fantasy; R, Reverse score*.

## Discussion

The analyses conducted resulted in the development of a scale comprised of a total of 16 items that converge separately on four factors. The first factor assesses one's ability to comprehend one's own and others' thoughts, beliefs, feelings, and perspectives (Waldinger et al., 2001). This ability involves being able to distinguish what individuals know about themselves in a given situation (how they think, feel, and behave) and what they know about others in that same situation (Ziv and Frye, 2003). Perspective taking is fundamental to the development of individuals' ability to interact meaningfully with other people, and to appropriate social functioning.

The second factor measures the personal distress experienced by the person in response to distress manifested by others or in emotionally stressful situations. Batson et al. (2002) defined personal distress, identifying it precisely as the experience of a negative emotional state (anxiety or worry) and leading to a self-oriented, selfish reaction or concern. Hoffman (1990) calls personal distress empathic overarousal and describes it as an involuntary feeling that occurs when the feeling shared by the observer becomes so pain-laden and intolerable that it becomes personal distress, leading the individual to withdraw from the situation. The personal discomfort response is similar to empathy, but differs from it because the emotion experienced by the observer is not necessarily attuned to that felt by the observed. From this perspective, it would appear that personal distress differs from empathy in that it is over-activated. Therefore, one could imagine that a particularly intense sharing experience causes an experience that is so strong that it is difficult to manage and, therefore, elicits personal discomfort. This empathic overarousal may affect the communication style of the person, who has difficulty containing his or her emotions and shows incapacity to mask emotional states in verbal interaction (Diotaiuti et al., 2020).

The third factor measures fantasy concerning the tendency to imagine oneself in fictitious situations. Empathic identification is mediated by the similarity that the reader/viewer more or less consciously recognizes between their own experiences and beliefs and those of the character. Thanks to this mechanism one participates in the story, one does not feel only an observer of events that happen outside. It is a complex phenomenon because in addition to the emotional component there is also a cognitive one, which consists in recognizing as one's own the conceptions of the world and the self to which the character is inspired.

The fourth factor measures the ability to feel concerned about someone and at the same time be able to offer concrete help in solving a problem or bringing comfort. It is therefore the most appropriate form of empathy because it coincides not only with the ability to understand the point of view of others (cognitive empathy) but at the same time to feel emotionally involved in what the other feels as pain, sadness, anger, fear, joy (emotional empathy) without being overwhelmed. This closeness or “right distance” allows one to offer concrete help so that the other “needy” person will take useful action to resolve their discomfort. Those who use this form of empathy feel the desire to do their best for the other person in order to alleviate their suffering and make themselves useful for their well-being. It is the most authentic form of empathy with which altruists are endowed.

Considering the correlations between the four factors, the study found that Perspective Taking had a robust association with Fantasy and Empathetic Concern, while the association with Personal Distress was lower and less significant. A similar trend result is also found in Ingoglia et al. (2016). The dominant focus on one's own emotional responses to contact with the negative emotions of others (Personal Distress) would probably result in a reduced capacity to empathize with the perspective of others. The study also found a lack of correlation between Empathic Concern and Personal Distress, proving that the latter could be read as a consequence of excessive and dysfunctional empathy, which activates an aversive rather than helpful emotional response to the person in difficulty (Fabi et al., 2019).

Convergent validity testing showed, as hypothesized, a strong association with prosociality, that is, the individual tendency to implement behaviors aimed at obtaining positive and beneficial effects on other people. When we talk about prosociality, we refer to various aspects related to helping, caring, sharing, cooperating and feeling solidarity. Prosociality, therefore, is not so much a unitary behavior, but rather a set of different behaviors; these can be guided by very different motivations and can translate, in practice, into physical help, verbal support, listening. The correlation that emerged between prosociality both with the general IRI index and with the subscales suggests that empathic dimensions have a significant antecedent role for altruistic activation.

This result is in line with findings from previous studies (Caprara and Pastorelli, 1993; Bandura et al., 1996). In our study, among the empathic dimensions, the strength of the association was greatest in the case of Perspective Taking. This appears to confirm what has already been found by Fuentes et al. (1993), Cigala et al. (2015), and Fang et al. (2019). Equally robust are the associations found between prosociality and the dimensions of Empathic Concern and Imagination. The association with Personal Distress was significantly lower, confirming the idea that focusing on one's own emotional responses hinders prosocial activation (Preston and Hofelich, 2012; Zaki, 2014). Compared to the initial hypotheses of convergence, it did not result in a significant (inverse) association with the global measure of Alexithymia, but rather with the EOT subdimension i.e., Externally Oriented Thinking. EOT refers to a specific tendency to deal with superficial themes and to avoid affective thinking (Franz et al., 2008). As Alexithymia is associated with poor socio-affective skills, some authors hypothesized alexithymic people have difficulties interacting and dealing with their social environment (Vanheule et al., 2007; Meganck et al., 2013) and demonstrate cold and distant social functioning as well as detachment from others, expecially in high alexithymia scorers. As can be observed in Table 6, in our study EOT correlated significantly inversely with both total IRI, PSA, and the empathic dimensions PT, EC, F, but not PD. While PD showed a negative relationship with DIF (Difficulty Identifying Feeling) and DDF (Difficulty Describing Feelings). The relationship between empathy and alexithymia has already been reported in the study by Guttman and Laporte (2002) who showed that (1) the IRI PD and PT scores were associated with DIF scores, (2) the IRI PD and EC scores were associated with DDF scores, (3) the IRI PT, F and EC scores were associated with EOT scores. More recently Grynberg et al. (2010) also conducted a study on the relationship between empathy and alexithymia, where EOT showed the same associations with the components of IRI reported by us. The presence in the individual of an emotionally poor thinking, superficially oriented mostly to external events, people or places, and basically unable to reach an introspective awareness is an individual characteristic that strongly limits the empathic abilities and the possibility to produce behaviors oriented to the protection and wellness of others (Eisenberg et al., 2010; Lockwood et al., 2014). It would be appropriate to further investigate the possible mediating role of EOT on the relationship of influence of empathy on prosocial behaviors (Spataro et al., 2020). Some recent contributions have also emphasised aspects related to temporal experience showing that individuals with manifest alexithymia are characterized by a high tendency to focus on negative aspects of the past and interpret the present fatalistically. This suggests that difficulties in identifying and describing feelings and emotions are associated with a negative bias for past and present events (Barchetta et al., 2021; Diotaiuti et al., 2021c).

Another goal of our study was to look at Measurement Invariance by Gender on the brief IRI's four-factor structure in order to further validate the measure and then screen for gender. The tool's measurement of invariance with respect to the gender factor confirmed the strong gender invariance and the excellent fit of the four-dimensional model for male and female.

The analysis of measurement invariance with respect to gender revealed important aspects related to potential gender differences in the person's experience of interpersonal responsiveness. The comparison of the values of the latent averages in the factors composing the brief IRI instrument showed that among the participants in our study, women reported values indicating a greater experience of Perspective Taking, Personal Distress, Fantasy, Empathic Concern than men. In particular, significantly higher values of personal distress can also be noted in women. The latter result agrees with the findings in Ingoglia et al. (2016).

There are numerous scientific studies that agree that females are able to “put themselves in the other person's shoes” more easily than males (Preston and De Waal, 2002; Archer, 2019; Benenson et al., 2021). As well as widespread is the belief that empathy can be considered a facilitator of prosocial behavior based on the assumption that emotional activation, generated by affective sharing of what another person is feeling, prompts the observer to engage in positive social behaviors such as lending help, giving comfort, sharing material goods. However, this belief, despite having received unanimous support in the past, is now accepted more critically (Underwood and Moore, 1982; Eisenberg, 1986; Batson, 1991; Batson et al., 2015). Several authors conclude that the relationship between empathic ability and prosocial behavior is not direct or necessary and that some other factors, such as individual differences or cognitive styles, must be taken into account, which can be held responsible for the development of this association (Davis, 2015).

In relation to individual differences, Christov-Moore et al. (2014) has shown that women engage in pro-social behavior more frequently and intensively than men (e.g., by spending more hours volunteering and making larger donations at fundraisers for the needy). However, this does not mean that women are the only ones who help others. Men do engage in altruistic behavior, but they do so in different contexts and with different resources than women. More specifically, women are more likely to engage in activities with a low risk of negative health consequences (low-risk-low-physical-strength), such as organizing food collections for charities. Men, on the other hand, prefer situations with a higher risk of negative consequences (high-risk-high-physical-strength), such as helping a person in danger of dying, or simply a friend during a move, by carrying heavy furniture.

In addition to the way they help others, men and women also differ in their choice of whom to help. Women tend to be more open to helping others, whether they are acquaintances or strangers, whereas men prefer to invest their resources in helping family and friends (George et al., 1998).

Comparing the brief version presented in this study with the brief version of the IRI proposed by Ingoglia et al. (2016), the four-factor structure is confirmed; the PT scale includes one more item (item 9, see Table 9) and reported slightly better internal consistency values; the EC scale confirmed the reverse direction of the original form, compared to the version of Ingoglia et al. (2016), in which instead the content direction was changed in a positive direction. Our version includes one more item (item 16, see Table 9. For this scale, the comparison with respect to internal consistency measures showed substantially equivalent values between the two version briefs. For the PD scale, the number of items is the same, but the content of item 6 is different: in our version, is included item 10 of the 28-scale (Albiero et al., 2006), in which the subjective response (of perceived vulnerability, “I feel helpless”) of the individual to (general) emotionally highly engaging situations is emphasized; whereas in the version of Ingoglia et al. (2016), item 27 is included, in which the content refers to emotional/cognitive breakdown (“I go to pieces”) in a situation where someone else urgently needs help. Comparison of internal consistency measures also reported slightly better values on this scale in our version. Regarding the Fantasy scale, our version does not include item 5 from the 28-version, which is present in Ingoglia et al. (2016) scale instead. The internal consistency measure was found to be essentially equivalent here between the two brief versions.

This study's findings should be viewed in light of several limitations. First, the IRI is a self-report measure of empathy that may be influenced by social desirability biases. Thus, it would have been beneficial to include other measures of empathy or social desirability in order to investigate potential bias associated with self-report measures, as well as test other aspects of the measure's validity, such as discriminant validity. Second, the sample is limited to two convenience Italian samples, which limits the study's generalizability. Finally, due to the cross-sectional nature of this study, we were unable to investigate the stability and evolution of the IRI's four-factor and higherorder structures over time. Future longitudinal research could account for these limitations while also testing developmental trends in empathy and providing a basis for comparison with these cross-sectional findings. Furthermore, future research should consider studying the MI of the IRI across cultures in order to validate the structure's comparability.

## Conclusion

This Italian validation study of a Brief Interpersonal Reactivity Index has shown good results with both the CFA and the Measurement Invariance across gender. This study added to the evidence that there is a link between alexithymia and a lack of empathy. In particular, the significant relationship between the presence of external oriented thinking and a low level of empathy has been highlighted. The measurement with the IRI-B could be useful for this predictive value in order to identify those people who manifest a mode of superficial thinking, tending to lack of imaginative and emotional processing, strongly oriented to external reality rather than introspection. Research has shown that, especially in adolescence, such alexithymic components have been associated with various forms of addiction, eating disorders, post-traumatic disorders, abuse and violence suffered (Larsen et al., 2006; Dalbudak et al., 2013; Petruccelli et al., 2014; Strickland et al., 2017; Garofalo et al., 2018; Gillespie et al., 2018; Leshem et al., 2019). In particular, it is necessary to undertake early (after the assessment) with these individuals psycho-educational interventions aimed at improving emotional awareness and affective regulation, in order to allow them to perceive the real state of need related to the emotion experienced and activate the ability to ask for help, comfort, closeness as well as to empathize and understand the feelings and needs of others. A second important contribution of the study is the strong association that emerged between empathy and prosocial behavior, which emphasizes the function of empathy as a motivating element of prosocial behavior (Sanmartín et al., 2011; Carter and Ellis, 2016; Diotaiuti et al., 2021a,b). In line with other studies, we could hypothesize that the assessment of empathy with the tool proposed here could therefore be used for predictive purposes in the evaluation of staff working in the field of caring (nurses, social workers, health workers, etc.) to assess their real willingness to act promptly to help and support users in need and bearers of difficulty.

## Data Availability Statement

The raw data supporting the conclusions of this article will be made available by the authors, without undue reservation.

## Ethics Statement

The studies involving human participants were reviewed and approved by Institutional Review Board of the University of Cassino and Southern Lazio. The participants provided their written informed consent to participate in this study.

## Author Contributions

PD, GV, and SM designed the study, analyzed the data, and discussed the results. PD, AG, and GV drafted the manuscript. AC and SM revised the manuscript. Finally, the authors have agreed to be accountable for all aspects of the manuscript in ensuring that questions related to the accuracy or integrity of any part of it are appropriately investigated and resolved.

## Conflict of Interest

The authors declare that the research was conducted in the absence of any commercial or financial relationships that could be construed as a potential conflict of interest.

## Publisher's Note

All claims expressed in this article are solely those of the authors and do not necessarily represent those of their affiliated organizations, or those of the publisher, the editors and the reviewers. Any product that may be evaluated in this article, or claim that may be made by its manufacturer, is not guaranteed or endorsed by the publisher.
